# CubeSat constellations provide enhanced crop phenology and digital agricultural insights using daily leaf area index retrievals

**DOI:** 10.1038/s41598-022-09376-6

**Published:** 2022-03-28

**Authors:** Kasper Johansen, Matteo G. Ziliani, Rasmus Houborg, Trenton E. Franz, Matthew F. McCabe

**Affiliations:** 1 Hydrology, Agriculture and Land Observation Group, Water Desalination and Reuse Center, Biological and Environmental Science and Engineering (BESE), King Abdullah University of Science and Technology, Thuwal, 23955-6900 Kingdom of Saudi Arabia; 2 Hydrosat S.à r.l., 9 Rue du Laboratoire, 1911 Luxembourg, Luxembourg; 3 Planet Labs Inc., Calibration, Analysis Ready Data, and Interoperability Operations (CARDIO), San Francisco, CA 94107 USA; 4 Institute of Agriculture and Natural Resources, School of Natural Resources, University of Nebraska-Lincoln, Lincoln, NE 68583-0973 USA

**Keywords:** Optical imaging, Plant sciences

## Abstract

Satellite remote sensing has great potential to deliver on the promise of a data-driven agricultural revolution, with emerging space-based platforms providing spatiotemporal insights into precision-level attributes such as crop water use, vegetation health and condition and crop response to management practices. Using a harmonized collection of high-resolution Planet CubeSat, Sentinel-2, Landsat-8 and additional coarser resolution imagery from MODIS and VIIRS, we exploit a multi-satellite data fusion and machine learning approach to deliver a radiometrically calibrated and gap-filled time-series of daily leaf area index (LAI) at an unprecedented spatial resolution of 3 m. The insights available from such high-resolution CubeSat-based LAI data are demonstrated through tracking the growth cycle of a maize crop and identifying observable within-field spatial and temporal variations across key phenological stages. Daily LAI retrievals peaked at the tasseling stage, demonstrating their value for fertilizer and irrigation scheduling. An evaluation of satellite-based retrievals against field-measured LAI data collected from both rain-fed and irrigated fields shows high correlation and captures the spatiotemporal development of intra- and inter-field variations. Novel agricultural insights related to individual vegetative and reproductive growth stages were obtained, showcasing the capacity for new high-resolution CubeSat platforms to deliver actionable intelligence for precision agricultural and related applications.

## Introduction

Growing populations in the developing world combined with shifting dietary patterns globally^1^ are driving projections of future food production increases well beyond current levels^2^. Precision agriculture will play an important role in delivering this increased food demand, requiring an additional level of agricultural intensification on an already constrained system^3^. At its heart, precision agriculture is essentially an effort to optimize agricultural production inputs to maximize outputs^4^. The optimization process is based on a range of data resources, spanning in situ sensing technologies as well as information derived from remote sensing, including ground robotics, unmanned aerial vehicles, and airborne and satellite image data^5^. Data streams, often analyzed in near real-time, provide information facilitating improved and timely farm management practices designed to enhance productivity in a more sustainable manner^6^. Information on crop phenology is particularly important for optimal timing of treatment applications^7^, which in turn can help harvest and yield optimization, as well as for forward planning of on-farm logistics^8^. For larger or spatially distributed fields, satellite imagery is often the only feasible means of deriving consistent and accurate information that is needed for intra-field spatiotemporal assessment of crop growth variability and performance^9^.

The identification of within-field variability is key to delivering upon the promise of precision agriculture. Amongst a range of supporting technologies, this effort has been accelerated by the relatively recent emergence of constellations of small light-weight CubeSats^10^. The most recent manifestation of these new Earth observing platforms provides a capacity for near daily global terrestrial coverage at an unprecedented resolution of approximately 3 m^11^. The emergence of this new observational platform has driven an increasing range of precision agricultural applications that have delivered insights into crop development and phenological events. For example, Aragon et al.^12,13^ estimated crop-water use from a time-series of CubeSat imagery, finding good agreement against ground-based assessments. Paul et al.^14^ used CubeSat images for detection of intermittent ponding in maize and soybean fields, while Sadeh et al.^15^ employed a sequence of CubeSat data to detect sowing dates, achieving an accuracy of fewer than 2 days. From a soil and vegetation perspective, Cai et al.^16^ used weekly CubeSat data for successful detection of nitrogen stress in corn, while Houborg and McCabe^11,17^ as well as Kimm et al.^18^ have demonstrated retrievals of leaf area index (LAI), finding that CubeSat data provided improved estimates relative to a fused MODIS-Landsat collection.

Leaf area index is an important biophysical variable for agricultural monitoring of crop physiological and phenological status, as well as for regular monitoring of yield potential and crop health^19,20^. Defined as the ratio of the one-sided foliage area to ground area, ground-based LAI measurements can be collected both indirectly and directly. Indirect measurements are often acquired optically based on the relation between vegetation indices and LAI, or via calculations of gap fraction and light transmission^21,22^. Direct measurements generally consist of destructive sampling and analysis of collected leaves. Although being both labor-intensive and time-consuming, direct LAI measurements are considered the most accurate^23^, but are impractical for operational purposes. In order to provide LAI estimates at the field scale and beyond, remote sensing-based crop-specific radiative transfer inversion models have also been applied^24^. Alternatively, field-based measurements may be scaled up using remotely sensed image data, or employed for independent calibration and validation purposes.

Indeed, image-derived LAI measurements have been used extensively in agricultural applications. For instance, LAI has been used for determining nitrogen fertilizer applications for maize production^25^, mapping chlorophyll content^26^, and estimating yield^27^. Image-based LAI retrievals have been performed at local, regional, and global scales. Daily LAI retrievals produced from different course resolution image sensors such as VEGETATION^28^, MODIS^29^, and AVHRR^30^ have provided useful information for evaluating temporal variations in vegetation cover and phenology at global scales, albeit not at a spatial resolution suitable for detailed assessment of individual agricultural fields. Course resolution MODIS-derived LAI data have also been used as reference data for producing MODIS-consistent LAI products from Landsat imagery at 30 m resolution [e.g.^31–33^]. While 30 m Landsat and 10 m Sentinel-2 based LAI products^34–36^ can be produced routinely, neither of these products provide the combination of spatial and temporal resolution required for precision agricultural applications. Using a non-continuous time-series of CubeSat-derived LAI, Houborg and McCabe^11^ demonstrated the spatiotemporal advantages in monitoring alfalfa when compared to 30 m resolution Landsat-8 data. In another study, Sadeh et al.^37^ fused cloud-free Planet CubeSat and Sentinel-2 imagery to produce surface reflectance and LAI information, achieving root mean square errors (RMSE) ranging from 1.73 to 1.78 based on CubeSat data over wheat fields in Australia and Israel using vegetation indices and empirical relationships. However, the applied fusion method was based on simple averaging of PlanetScope and Sentinel-2 data, potentially incorporating cross-sensor radiometric inconsistencies common to PlanetScope surface reflectance data, and used linear interpolation between dates with missing or cloud-affected data. Sadeh et al.^37^ reported that LAI values > 3 were underestimated without the application of a correction equation relying on field-based LAI measurements.

Until now, there has been no demonstration of the capacity to retrieve consistently calibrated daily-scale high-resolution time-series of crop structural parameters such as LAI throughout an entire growing season. Here, we demonstrate that capacity for one rain-fed and two irrigated fields of maize in Nebraska, USA and show how daily CubeSat-derived LAI measurements produced using a multi-satellite image fusion approach can be related to plant phenology. In addition to providing spatiotemporally dense information that can be used to drive treatment and irrigation scheduling, identification of within-field variability, and monitoring of crop phenology and health, these data may deliver further insights to facilitate food production and early estimates of yield^38,39^.

## Materials and methods

### Study site and field campaign data

**Figure 1:**
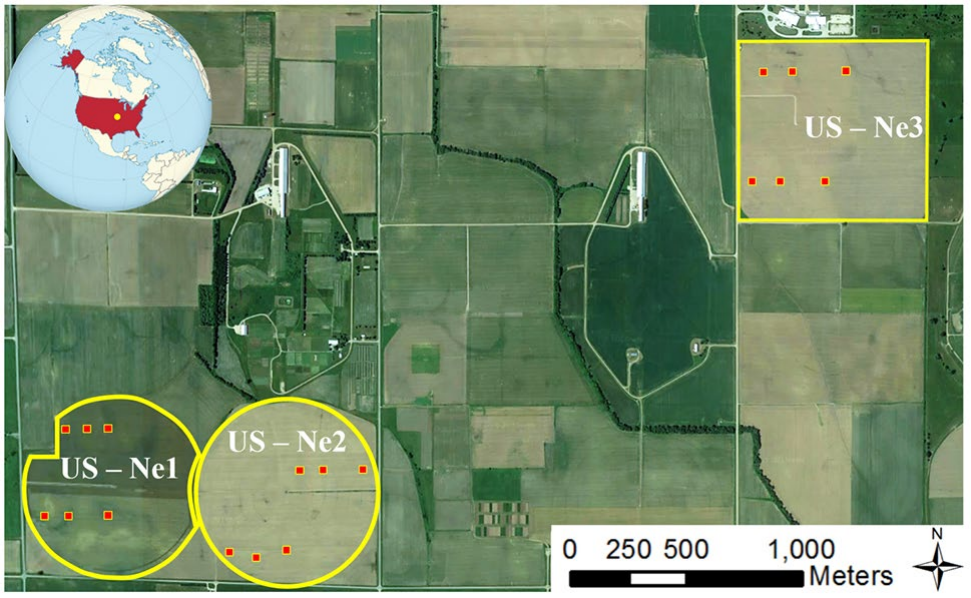
Locations of the three studied maize fields in Nebraska, USA displayed in a true color composite of Maxar imagery from Google Earth collected on June 5, 2018 (Google, Imagery ©2022 Maxar Technologies, U.S. Geological Survey, USDA Farm Service Agency, Map data ©2022). These included irrigated fields US-Ne1 and US-Ne2 and the non-irrigated US-Ne3 field (yellow outlines). The red squares identify each of the intensive measurement zones, where plants were collected for LAI measurements. Software used to produce the map: ArcGIS version 10.8.1 (www.esri.com/en-us/arcgis).

The study sites were located within the domain of the Eastern Nebraska Research and Extension Center (ENREC) at the University of Nebraska-Lincoln in the United States. Three separate fields growing yellow maize for cattle feed were examined during the 2019 growing season, with two irrigated fields covering an area of 48.7 ha (US-Ne1) and 52.4 ha (US-Ne2), and a third rain-fed field covering an area of 65.4 ha (US-Ne3)^40^ (Fig. 1). US-Ne1, Ne2 and Ne3 were planted on April 19, 23 and 24, respectively. Approximately one week prior to planting, nitrogen (N) fertilizer was applied at a rate of 175 kg N ha^-1^ for US-Ne1, and 157 kg N ha^-1^ for US-Ne2 and Ne3, by coulter injection of liquid urea ammonium nitrate. In addition to this pre-planting application, a further 45 kg N ha^-1^ was used for the irrigated sites at the beginning of July to improve maize N use efficiency. US-Ne1 received approximately 7 mm of irrigation on April 23, June 13, and July 1, 2019, followed by approximately 31 mm of irrigation on July 15, 24 and 29 and August 9. US-Ne2 received approximately 31 mm of irrigation on July 2, 8, 15, and 29. Herbicides and pesticides were applied in accordance with standard practices prescribed for maize production in eastern Nebraska^41^. Monthly rainfalls recorded after planting were 20, 147, 111, 57, 72, 107, and 128 mm between April 23 and October 31, respectively, with radiation totaling 3324 MJ/m^2^ for the same period.

LAI was collected from each field within six 20 × 20 m plots (referred to herein as intensive measurement zones; IMZs), which were established to perform detailed plant measurements^40^. Assessment of the phenological stage and destructive samplings of 5–7 plants per IMZ were carried out 13 times, 1–2 weeks apart, between May 23 and September 25 for US-Ne1 and Ne2, and 11 times between May 29 and September 18 for US-Ne3. Maize plants used for LAI measurements were collected following guidelines provided by the University of Nebraska-Lincoln at the Eastern Nebraska Research and Extension Center. This project is part of the Ameriflux Network and Long Term Agricultural Research Network. In August, sampled plants averaged 2.81 m in height (with a range of 2.46–3.08 m), 2.56 m (1.82–2.78 m), and 2.38 m (1.78–2.82 m) for US-Ne1, Ne2, and Ne3, respectively. The sampled plants were subsequently analyzed in a laboratory and divided into stems, reproductive organs, and green and dead leaf components for leaf area and biomass estimation^42,43^. The leaf area was then measured with a LiCOR LI-3100C area meter. Herein, only the green leaf components were used for calculating LAI and for comparison with the image-derived measurements.

### Production of planet CubeSat-based LAI

Planet CubeSat data consisting of blue (450–510 nm), green (510–530 nm), red (640–670 nm), and near-infrared (NIR; 850–880 nm) bands were acquired from the three PlanetScope constellations (Dove Classic, Dove Refreshed, SuperDove) over the study site (Fig. 1) from April 1 to November 30, 2019. A total of 159 CubeSat images, with 52 of these affected by clouds or cloud shadows, underwent Planet Fusion processing^44^, which leverages data from multiple sensor sources, including three PlanetScope generations, Sentinel-2, Landsat 8, Visible Infrared Imaging Radiometer Suite (VIIRS), and Moderate Resolution Imaging Spectroradiometer (MODIS). The fusion of these multi-satellite platforms combines uniquely different radiometry, quality, and resolution characteristics to produce analysis-ready surface reflectance data that inherit the best traits from each sensor while ensuring radiometric consistency across the fleets. Houborg and McCabe^45^ developed the CubeSat Enabled Spatiotemporal Enhancement Method (CESTEM) to enable radiometric harmonization of CubeSat time-series data with Landsat-8 and Sentinel-2 data. In fact, Kong et al.^46^ found that normalized difference vegetation index (NDVI) values derived using the CESTEM approach provide better agreement with in situ NDVI measurements than other fusion approaches such as the Enhanced Spatial and Temporal Adaptive Reflectance Fusion Model (ESTARFM)^47^, the Flexible Spatiotemporal DAta Fusion model (FSDAF)^48^, and SaTellite dAta IntegRation (STAIR)^49^. For the Planet Fusion processing, CESTEM^45^ was used to rigorously harmonize multi-sensor spectral data into a consistent radiometric surface reflectance standard for full fleet interoperability. The Framework for Operational Radiometric Correction for Environmental Monitoring (FORCE)^50^ was used to generate a combined Landsat 8 and Sentinel-2 BRDF-adjusted surface reflectance product (30 m) to represent the “gold standard”. Planet Fusion processing (Planet version 1.0.0-beta.1 used herein) also included: (1) sub-pixel geometric alignment of source imagery; (2) temporally driven cloud and cloud shadow detection; (3) fusion of Sentinel-2 and Landsat 8 data to fill gaps in PlanetScope coverage; and (4) advanced gap-filling based on multi-sensor observation data both before and after the prediction date, delivering daily gap-free 4-band radiometrically robust and spatiotemporally consistent surface reflectance data.

**Figure 2:**
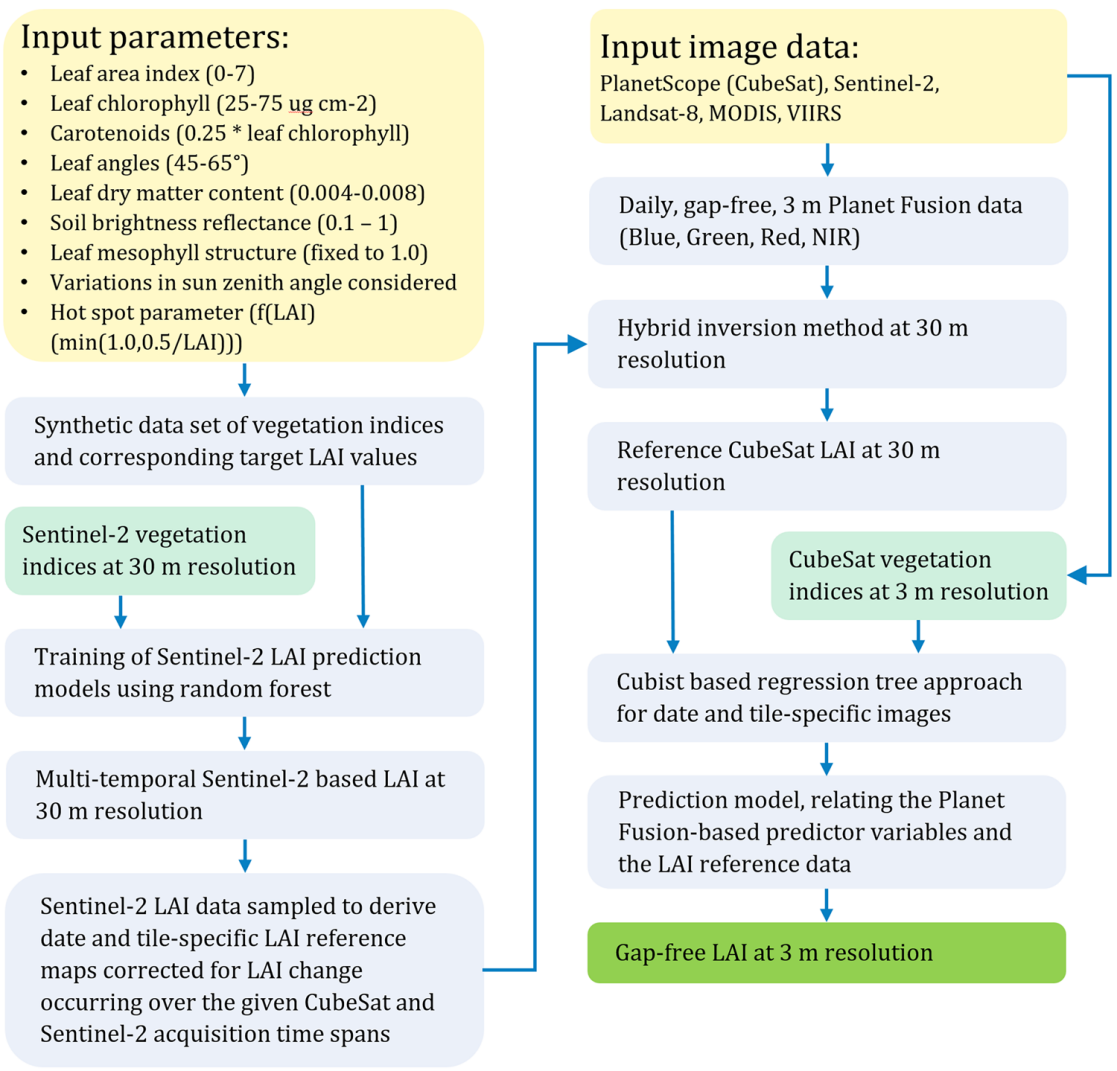
Flowchart of the processing workflow to produce daily gap-free LAI maps at 3 m resolution. The forward runs of PROSAIL produces a dataset of vegetation indices and corresponding LAI values, which are used to train the Sentinel-2-based LAI prediction models using random forest. The models are applied to the Sentinel-2 vegetation index data to produce Sentinel-2-based LAI at 30 m. The multi-temporal Sentinel-2 LAI data are then sampled to derive date and tile-specific LAI reference maps corrected for LAI change occurring over the given CubeSat–Sentinel-2 acquisition time spans. The relative LAI change is derived on the basis of a simple multi-variate regression model trained on the multi-day pool of Sentinel-2-LAI and co-incident Planet Fusion vegetation index predictor data. Finally, date and tile-specific model learning (Cubist) and prediction is performed by relating the Planet Fusion-based predictor variables and the LAI reference data.

The Planet Fusion derived surface reflectance data (daily, gap-free, 3 m) were used as the foundation for deriving LAI across the three maize fields. LAI was retrieved using a novel hybrid inversion method (Planet version 1.0.0-alpha.1 used herein) that combines non-parametric machine-learning and state-of-the-art physically-based understanding. The adopted model training and prediction approach, which was independent of the ground-collected LAI, leverages (1) the enhanced spectral capability of the Sentinel-2 sensor data to establish robust spectral-to-plant trait relationships via machine learning, and (2) 4-band CubeSat data to enable significant enhancements both spatially and temporally (Fig. 2). As part of the Planet Fusion surface reflectance product generation, Sentinel-2 (and Landsat-8) surface reflectance data were processed and harmonized using the FORCE atmospheric correction approach with an output at 30 m resolution. Resampling to a coarser resolution (30 m) optimized the Sentinel-2-based LAI generation and CubeSat-Sentinel-2 fusion. Therefore, Sentinel-2 based LAI at 30 m was estimated on the basis of random forest prediction models trained on a comprehensive synthetic dataset of target (i.e., LAI) and explanatory spectral variables (i.e., vegetation indices). The training dataset was constructed from forward runs with the PROSAIL^51^ canopy reflectance model over a wide distribution of soil brightness levels (e.g. soil reflectance), leaf (e.g., leaf pigment concentrations), and canopy (e.g., LAI, leaf angle) parameter realizations (Fig. 2). The explanatory dataset consisted of nine Sentinel-2 bands transformed into a broad set of vegetation indices (Table 1) covering the visible (V), red-edge (RE), near-infrared (NIR), and shortwave infrared (SWIR) domain in order to learn robust non-linear vegetation index—LAI associations applicable to a wide range of surface characteristics and LAI values. While the high spectral resolution of Sentinel-2 helps mitigate the ill-posed nature of canopy reflectance model inversion (i.e., the fact that different combinations of model variables can produce near-identical surface reflectance spectra), prediction models with different spectral feature sets (i.e., VNIR + RE, VNIR + SWIR, VNIR + RE + SWIR) were combined as an additional regularization constraint to improve robustness and reduce the overall uncertainty of the LAI predictions. A model based only on the 4-band CubeSat data would hinder the capacity to capture subtle variations and the full range of LAI magnitudes. Incorporating Sentinel-2 bands in the red-edge and SWIR portions of the spectrum significantly helps in constraining the LAI retrieval process and increases the sensitivity to LAI, providing enhanced performance over denser canopies^17,52^.

**Table 1:** List of vegetation indices across the visible (V), red-edge (RE), near infrared (NIR), and shortwave infrared (SWIR) domains used in the training of the random forest (Sentinel-2) and Cubist (Sentinel-2 and Planet Fusion) based leaf area index prediction models.

Vegetation index	Abbre-viation	Equation	Spectral category	Source
Simple ratio	SR	$b_{865}/b_{665}$	V, NIR	S2, PF
Normalized difference vegetation index	NDVI	$\left( {b_{865} - b_{665} } \right)/\left( {b_{865} + b_{665} } \right)$	V, NIR	S2, PF
Optimized soil adjusted vegetation index	OSAVI	$1.16 \times \left( {b_{865} - b_{665} } \right)$/$\left( {b_{865} + b_{665} + 0.16} \right)$	V, NIR	S2, PF
Green simple ratio	GSR	$b_{865}/b_{560}$	V, NIR	S2, PF
Green normalized difference vegetation index	GNDVI	$\left( {b_{865} - b_{560} } \right)/\left( {b_{865} + b_{560} } \right)$	V, NIR	S2, PF
Modified triangular vegetation index 2	MTVI2	$1.5 \times \frac{{\left( {1.2 \times \left( {b_{865} - b_{665} } \right) - 2.5 \times \left( {b_{665} + b_{560} } \right)} \right)}}{{\sqrt {\left( {2 \times b_{865} + 1} \right)^{2} - \left( {b_{865} - 5 \times \sqrt {b_{865} } } \right) - 0.5} }}$	V, NIR	S2, PF
Enhanced vegetation index 2	EVI2	$2.5 \times \left( {b_{865} - b_{665} } \right)/\left( {b_{865} + 2.4 \times b_{665} + 1} \right)$	V, NIR	S2, PF
Modified chlorophyll absorption ratio index	MCARI	$\left( {\left( {b_{704} - b_{665} } \right) - 0.2 \times \left( {b_{704} - b_{560} } \right)} \right) \times \left( {b_{704} /b_{665} } \right)$	V, RE	S2
MERIS terrestrial chlorophyll index	MTCI	$\left( {b_{740} - b_{704} } \right)/\left( {b_{704} - b_{665} } \right)$	V, RE	S2
Vogelmann red edge index	VREI1	$b_{740}/b_{704}$	RE	S2
Red edge normalized difference vegetation index	RENDVI	$\left( {b_{865} - b_{704} } \right)/\left( {b_{865} + b_{704} } \right)$	RE, NIR	S2
Red edge normalized difference vegetation index 2	RENDVI2	$\left( {b_{865} - b_{740} } \right)/\left( {b_{865} + b_{740} } \right)$	RE, NIR	S2
Reduced simple ratio	RSR	$b_{865}/b_{704}$	RE, NIR	S2
Reduced simple ratio 2	RSR2	$b_{865}/b_{740}$	RE, NIR	S2
Normalized Difference Water Index	NDWI	$\left( {b_{865} - b_{1614} } \right)/\left( {b_{865} + b_{1614} } \right)$	NIR, SWIR	S2
Normalized difference water index 2	NDWI2	$\left( {b_{865} - b_{2202} } \right)/\left( {b_{865} + b_{2202} } \right)$	NIR, SWIR	S2
Mid-infrared simple ratio	MSR	$b_{865}/b_{1614}$	NIR, SWIR	S2
Mid-infrared simple ratio 2	MSR2	$b_{865}/b_{2202}$	NIR, SWIR	S2
Plant senescence reflectance index	PSRI	$\left( b_{665} - b_{560} \right)/b_{704}$	V, RE	S2

The indicated spectral wavelengths represent the center of the corresponding Sentinel-2 band. Note that the four Planet Fusion bands were identical to the Sentinel-2 bands as the bands were aligned during the Planet Fusion radiometric harmonization approach.

While the random forest approach performs well for modeling complex and non-linear associations such as those produced by forward runs of PROSAIL, Cubist on the other hand (using a limited set of multi-variate regressions) is quick to train and build, while being less prone to overfitting when applied to unseen data (e.g. for modeling the temporal change in LAI between Sentinel-2 and CubeSat acquisitions)^17^. Similar to the Planet Fusion radiometric harmonization approach, Sentinel-2 LAI estimates serve as the “gold reference”, as outlined in^45^. CubeSat-based relative LAI change information (30 m) over given Sentinel-2-CubeSat acquisition time spans was derived using a set of multivariate regression models trained on the available multi-day pool of Sentinel-2 LAI and day-coincident CubeSat-based vegetation index predictor data (Table 1). With this information, Sentinel-2 LAI reference samples were drawn from multiple Sentinel-2 scenes, correcting for any change in LAI magnitudes, occurring over the acquisition time spans to provide weighted and outlier screened pixel-wise (30 m) estimates of reference LAI for any given CubeSat tile. The Sentinel-2-based LAI reference sampling was specific to each Planet Fusion tile as well as the date of that tile (Fig. 2). Any given Planet Fusion tile was associated with a corresponding Sentinel-2 LAI reference tile, which was based on LAI sampling from multiple Sentinel-2 LAI images in the past (or future, relative to the prediction date) acquired over the Planet Fusion tile domain. As a result, the models were highly localized (date and tile-specific) and therefore will not be affected by transferability issues. For a full Planet Fusion tile (8000 × 8000 pixels of 3 m), LAI data for 800 × 800 pixels (640,000 samples) were used for training. In order to map LAI at the spatial and temporal resolution of the CubeSat data, a Cubist rule-based regression tree approach was used to learn the relationships between CubeSat-based vegetation index predictor variables and the derived LAI reference data for any given CubeSat tile. The resulting date and tile-specific prediction models were then used to derive gap-free LAI at 3 m resolution, with the cadence (daily) of the CubeSat surface reflectance data. While the training was performed at 30 m, the CubeSat-based LAI at 3 m was achieved by applying the model to the vegetation index data at the 3 m level, with the Sentinel-2 data being matched to the Planet Fusion tile domain as part of the Planet Fusion processing^44^. As the training was date and tile-specific, very good fits (i.e. with an R^2^ > 0.95) were achieved between the Sentinel-2 based LAI reference data and the CubeSat-predicted LAI, with overfitting considered unlikely due to the constrained approach.

For each of the three maize fields, the in situ LAI measurements derived from the six IMZs (see Fig. 1) were used to evaluate the CubeSat-based LAI maps. The laboratory-derived LAI measurements of the green plant components of the 5–7 plants assessed per IMZ were averaged and then multiplied by the plant population within each IMZ to form a representative LAI value^19^. Using the center location of each 20 × 20 m IMZ, a window of 7 × 7 CubeSat pixels (i.e. 21 × 21 m) was then used to extract an average LAI value for comparison with the in situ LAI measurements. RMSE and relative RMSE (rRMSE) between in situ and CubeSat-derived LAI values were reported. With six IMZs for each of the three fields and 13 multi-temporal observations for US-Ne1 and Ne2 and 11 for US-Ne3, a total of 222 observations were available for comparison with the CubeSat-derived LAI maps for the corresponding dates.

## Results and discussion

### Evaluation of CubeSat-retrieved LAI

**Figure 3:**
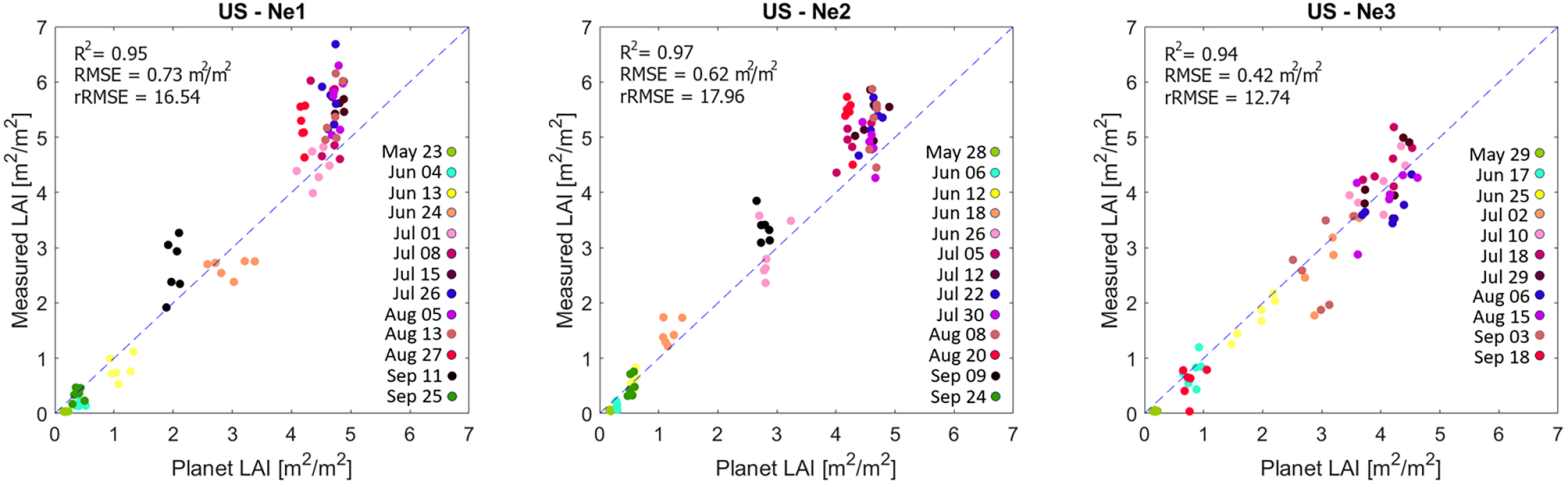
Scatterplots between field-measured LAI and LAI estimates of 7 × 7 pixels derived from the corresponding locations of the Planet CubeSat data collected 13 times during the growing season for US-Ne1 and Ne2 and 11 times for the US-Ne3 maize field.

Independent assessment of the LAI values derived from the CubeSat-based approach was performed against the field-derived LAI measurements. As can be seen in Fig. 3, there was a strong linear relationship between the field and CubeSat-based LAI measurements, producing coefficient of determination (R^2^) values > 0.94. Interestingly, the rRMSE for US-Ne3 (12.74) was significantly lower than those from the two irrigated fields (16.54 and 17.96; Fig. 3), suggesting more accurate LAI estimates within the rain-fed field. LAI values ≤ 5 generally followed a linear relationship, whereas the field-based LAI values > 5 were in most cases underestimated by the CubeSat-based approach; a common characteristic identified in other studies using satellite imagery for estimating LAI [e.g. 53,54]. While the laboratory-based measurements of LAI represented the green leaf cover, the CubeSat image data provide a top-down view, which may be affected by the leaf angle distribution, clumping effects, senescent leaves, plant stems, and any exposed soil background. Hence, variations between the two estimates can be expected, especially due to changing soil color and texture or the presence of weeds^55^. Canopy bi-directional reflectance, including that associated with canopy shadowing, may also affect image-derived vegetation structural measurements such as LAI^56^.

Remotely sensed vegetation indices generally employ a combination of two spectral bands, with these indices showing different levels of correlation with LAI, where correlations often vary as a function of the phenological crop stage^57,58^. Here, we integrated a number of vegetation indices, incorporating the visible, red edge, NIR and SWIR bands of Sentinel-2 for generating LAI reference data to optimize the detection of subtle variations and to capture the full range of LAI magnitudes. Using individual vegetation indices as an indicator of LAI has proven difficult in the past, due to sensitivities to background reflectance effects, differences in crop varieties and their structure, and variations in canopy shadowing^55,56^, all of which can hinder temporal comparison of (unitless) indices for phenology characterization. As such, quantitative measurements of biophysical parameters, such as LAI, tend to be more appropriate to compare across time and space^59^. However, previous studies have found that remotely sensed vegetation indices can saturate at high LAI values, i.e. become insensitive to variations in LAI values above a certain threshold, due to the horizontal leaf stratification^60^, which provides a likely explanation of the CubeSat-based LAI underestimation in fields US-Ne1 and 2 from the beginning of July to mid-August depicted in Fig. 3. As the rain-fed US-Ne3 field generally had LAI values < 5, saturation effects were therefore not a strong consideration. Sadeh et al.^37^ found fused Sentinel-2 and CubeSat data-derived LAI values > 3 of wheat fields to saturate, requiring model adjustments. After their model adjustment, R^2^ ranged from 0.27 to 0.92, with RMSEs of 0.35–0.63 depending on the vegetation indices used. Kimm et al.^18^ produced LAI approximations for corn with an R^2^ of 0.76 and a RMSE of 1.12 based on CubeSat data. As such, our retrieval accuracies are within or better than those reported by both Kimm et al.^18^ and Sadeh et al.^37^, and are similar or exceed LAI estimates from Sentinel-2 [e.g. 61,62] and Landsat [e.g. 63–65].

### Spatiotemporal LAI dynamics drive field insights

**Figure 4:**
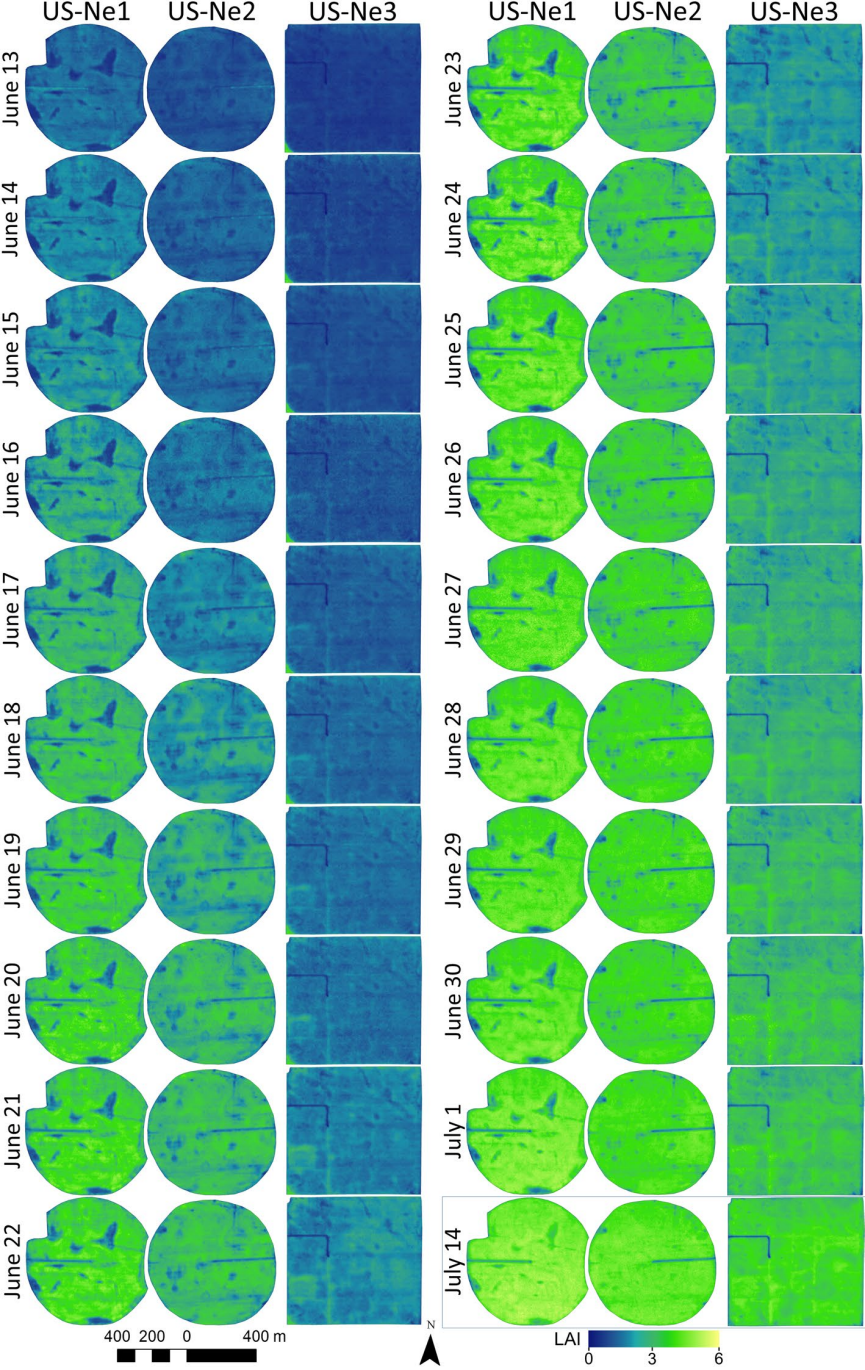
Daily sequence of leaf area index (LAI) maps, covering 19 days (June 13–July 1, 2019) during part of the vegetative stage and one selected day (14 July) with peak LAI values of the two irrigated US-Ne1 and Ne2 fields and the non-irrigated US-Ne3 field. Software used to produce the maps: ArcGIS version 10.8.1 (www.esri.com/en-us/arcgis).

The precise application of inputs such as irrigation, fertilizer, herbicides, and pesticides at the right time, rate and place within a field^66^ requires spatiotemporally dense data delivered with a low latency. Having a spatial resolution sufficient to identify intra-field variability, while obtaining daily temporal updates on the progression of plant growth, would provide a level of actionable intelligence that farmers could exploit to tailor management decisions for yield optimization^67,68^. High spatiotemporal resolution data also allows the consequences of management decisions and interventions to be assessed. For instance, fields US-Ne1 and Ne2 were planted on April 19 and 23, 2019, respectively. The 4-day time gap between planting of the two fields can be observed in Fig. 4, with the progression of LAI values in US-Ne2 consistently lagging those of US-Ne1. The non-irrigated US-Ne3 field that was planted on April 24 appears to be approximately 5 days behind the irrigated US-Ne2 field, even though there was only 1 day difference in planting and irrigation did not start until July 2 (see Fig. 4). Following irrigation, it becomes apparent that the rainfed US-Ne3 field had lower LAI than the two irrigated fields (e.g. 14 July in Fig. 4): an expected outcome given the differing volumes of water available to the crops. The relative temporal lag in LAI development between the three fields remained throughout the vegetative stage (Fig. 4).

Intra-field variability in LAI is evident across all three fields, with underperforming darker patches easily recognizable during the greening period displayed in Fig. 4 (June 13-July 1), and even at the very beginning of plant growth (June 13). While these are most likely the imprint of underlying soil characteristics, irrigation issues, or inconsistencies during planting^69^, the spatially dense CubeSat retrievals provide detail that would likely be unresolved using coarser scale satellite platforms such as Landsat^11^ and perhaps even Sentinel-2^70^. The full resolution (i.e., 3.125 m) of the CubeSat LAI maps permits an improved delineation of field sections showing optimal or suboptimal growth performance. The advantage of the daily image sequence is particularly pronounced given the rapid crop development during the vegetative stage of maize. It is noticeable at the time of peak LAI values (July 14) that the underperforming patches during the vegetative stage tend to dissolve and reach LAI values similar to the remaining parts of the fields, particularly in the US-Ne2 field, which received two lots of 31 mm irrigation between July 1–14.

**Figure 5:**
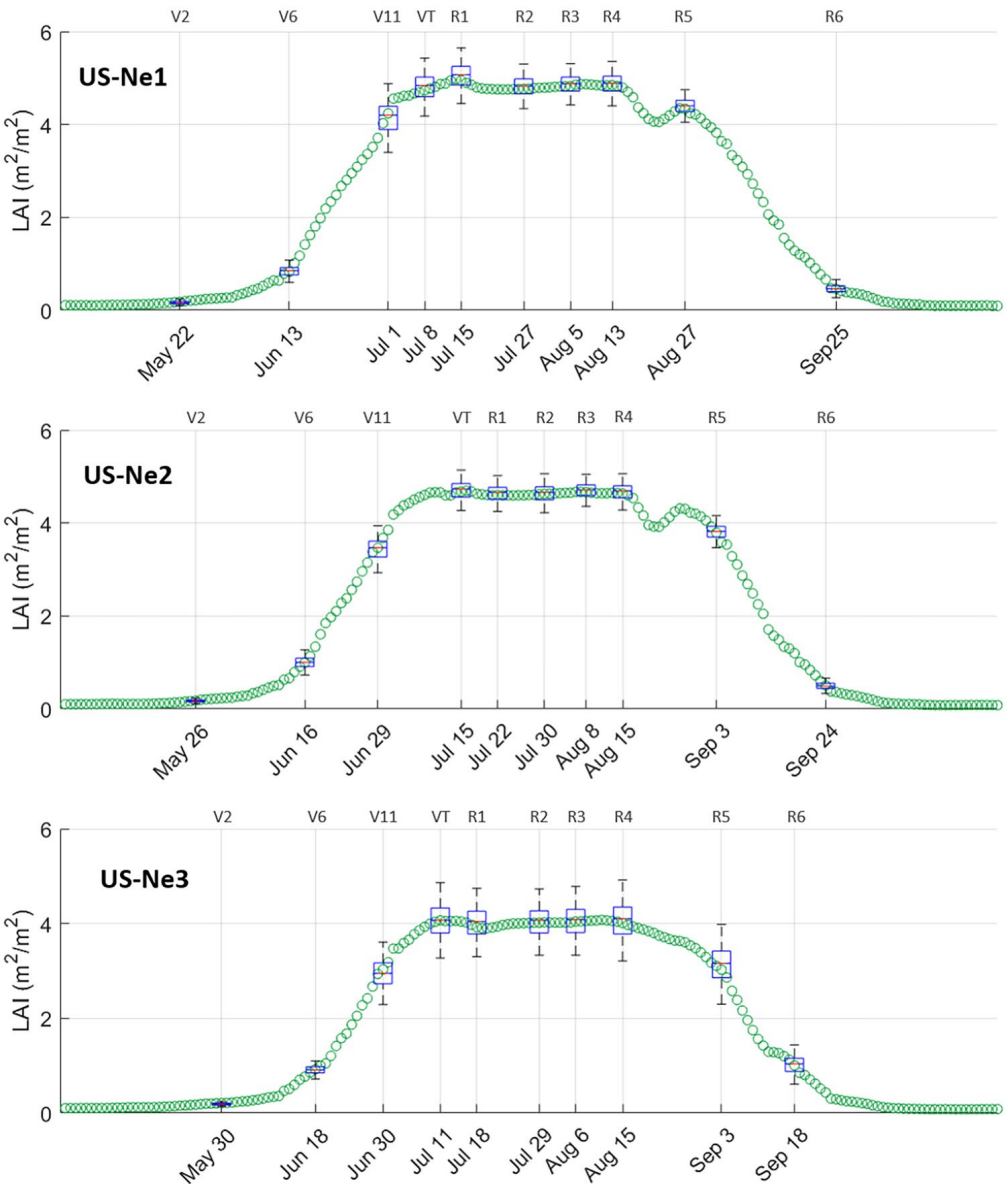
Box-and-whisker plots showing the field-identified stages of the 2nd (V2), 6th (V6) and 11th (V11) leaf collar, tasseling (VT), and the six reproductive stages, representing silking (R1), blister (R2), milk (R3), dough (R4), dent (R5) and maturity (R6). Boxes: interquartile range (IQR); lower limit of boxes: first quartile (Q1); upper limit of boxes: third quartile (Q3); whiskers: Q1-1.5(IQR) and Q3 + 1.5(IQR); line through boxes: median. All outliers were removed for visual clarity. Each green circle represents the average daily CubeSat-derived LAI per field during the growing season.

Identifying different growth stages of maize is a key component for precision insights, as plants have different requirements at different phenological stages^7^. To investigate the relation of CubeSat-derived LAI with key phenological stages of maize, box-and-whisker plots were produced to show the range of LAI pixel values within each field at specific field-identified vegetative and reproductive growth stages (Fig. 5). At the early vegetative stage, representing the second leaf collar (V2) at the end of May, CubeSat-observed LAI values can be seen to increase. Subsequently, rapid LAI increases occur in response to plant growth and the production of additional leaf collars (e.g. V6 and V11). The vegetative stages see high nutrient uptakes and rapid plant growth, which relies on moisture, temperature and light interception^25^. Maximum LAI was reached around the time of tasseling (VT, at the beginning of July) and silking (R1, in mid-July), which is the first reproductive stage of maize, coinciding with plant growth that shifts towards pollination and kernel formation. Plant stress (nutrient and moisture deficiencies) prior to tasseling and during pollination can greatly affect pollination and yield, emphasizing the need for careful management inputs in relation to maize phenology^71^. Based on Fig. 5, the tasseling stage was clearly identified as the peak, with a subsequent plateau in LAI, demonstrating the capacity to use daily LAI information for fertilizer and irrigation scheduling.

During the first four reproductive stages of kernel development, i.e. silking (R1), blister (R2), milk (R3) and dough (R4), no distinct variation in LAI was observed, despite the continuing increase in reproductive biomass. During kernel development, moisture stress, coupled with high temperatures, nutrient deficiency, disease or insect attack can significantly reduce kernel size and yield^71^. Hence, sudden drops in LAI values during the initial reproductive stages may be an indication of biotic or abiotic stress, requiring urgent management intervention. However, the spectral saturation at high LAI values may have contributed to the similarity in the observed LAI from the tasseling stage and during the first four reproductive stages, which may conceal fluctuations in LAI values above 5, especially in the irrigated fields. The LAI of maize fields depends on the planting density, plant variety, rainfall, temperature, sunlight, availability of nutrients and overall crop growth^72^. In some parts of the world, the LAI of maize is generally below 5^53,73–75^, which would eliminate saturation issues. Towards the dent stage (R5) at the end of August and beginning of September, the number of green leaves started decreasing, while the number of dead leaves increased, resulting in a distinct decrease in LAI. According to O’Keeffe^71^, the last irrigation should generally occur when the grain is well dented (a couple of weeks before maturity) to reach maximum yield, emphasizing the benefits of daily LAI information, as the R5 stage could clearly be identified in Fig. 5 for all three fields. The senescence of green leaves progressed rapidly towards the second half of September, when the plants reached physiological maturity (R6) and maximum biomass accumulation. Approximately four weeks prior to harvest (beginning of November), green LAI values approached zero, as all leaves had dried at that stage. As the yellow maize was used for cattle feed, it is often common practice to postpone harvest until all plants are fully dried for silo storage, hence highlighting another advantage of daily LAI information for harvest scheduling.

While the relation of maize phenology to the LAI time series (which covers the full growth cycle) provides useful agroinformatic insights, real-time forecasting of phenology based on a daily evolving time-series of LAI information during the growth cycle may be more difficult to interpret (e.g. it is difficult to determine when maximum LAI values are reached before seeing a plateau in the trend). However, based on a gradually evolving time-series of image-derived LAI values added with a 2–3 day latency, information suited for management practices can still be obtained. Such information includes steadily increasing LAI values during the vegetative stage, timing of silking based on a week of LAI plateauing, premature drops in LAI within the first month of LAI plateauing (potentially indicating some kind of biotic or abiotic stress), and full senescence of the field when green LAI values approach zero. In case of cloud cover, Planet Fusion tiles can generally be produced with a 48-h latency using the forward-fill operation^44^. However, prolonged periods of cloud cover may impact the accuracy of LAI predictions.

Not surprisingly, given the increased potential for plant stress due to water deficit, the rain-fed field (US-Ne3) displayed a larger range of LAI values compared to US-Ne1 and Ne2 in the hotter and drier months of July, August and September (Fig. 5). US-Ne3 also experienced a slightly delayed greening stage and started to senesce, reflected by a drop in green LAI at the end of August, before both US-Ne1 and Ne2. More generally, US-Ne3 displayed lower LAI values than both US-Ne1 and Ne2, indicating the increased production of biomass due to the irrigation of the other two maize fields. In fact, field measurements of biomass for the three fields at the time of plant physiological maturity (R6) showed a difference of approximately 7000 kg/ha between the irrigated (25,000 kg/ha) and the rain-fed (18,000 kg/ha) fields. Larger production differences between irrigated and rain-fed maize are likely to be more distinct in drier years, where the timing and amounts of irrigation can significantly impact end-of-season yield^76^.

## Conclusions

The provision of high resolution spatiotemporal information is central to realizing the precision agricultural advances needed to achieve global food and water security objectives. Providing farmers with timely information at the within-field scale is a significant step in this direction, and not only allows for the optimization of variable inputs, such as water and nutrients, but also for the early detection of areas with potentially low productivity. Such high resolution digital insights enable time-critical management intervention that can be used to facilitate yield optimization. Leveraging the high radiometric quality of Sentinel-2, Landsat-8, and VIIRS/MODIS time-series, a CubeSat-based LAI product was used to track within-field LAI variations across several maize fields through the course of an entire growing season. Apart from offering new spatiotemporal insights into crop development and behavior, these daily CubeSat-based LAI data demonstrated the capacity to capture recognized relationships between LAI and key vegetative and reproductive phenology stages. CubeSat-based LAI was produced with high accuracy (rRMSE and RMSE between 12 ando 18% and 0.42 and 0.73, respectively) and correlated highly with field-collected measurements. Some saturation of LAI values greater than 5 was noted, potentially contributing to LAI maps with reduced field heterogeneity at the tasseling stage and during the first four reproductive stages (R1-4). Importantly, a decrease in LAI values was recorded for all three fields during the dent (R5) stage, followed by a significant drop towards the plant majority stage (R6). More generally, the CubeSat data demonstrated clear capacity to reproduce key crop development stages and growth patterns from initial planting, to maturity and ultimately harvest. Ongoing work is required to demonstrate broader scale applications of these high spatiotemporal data assets. Research required to further advance the precision agricultural application of these novel CubeSat data might include an assessment of crop growth patterns, the influence of management decisions, an evaluation of retrievals over different crop varieties, or addressing yield prediction, disease detection and soil monitoring related challenges. Such analyses will help to determine the contribution of high spatiotemporal resolution information towards sustainable agricultural intensification and food production and better quantify the cost-to-benefit ratio of using high-resolution satellite-based daily products for precision agricultural applications.

## Data Availability

The Planet Fusion imagery and LAI data can be provided by KAUST pending scientific review. Requests for the data should be submitted to: Dr Kasper Johansen, kasper.johansen@kaust.edu.sa. All remaining data are presented in the paper.
